# A Simple Structure for an Independently Tunable Infrared Absorber Based on a Non-Concentric Graphene Nanodisk

**DOI:** 10.3390/ma15062296

**Published:** 2022-03-20

**Authors:** Kun Yu, Peng Shen, Wei Zhang, Xicheng Xiong, Jun Zhang, Yufang Liu

**Affiliations:** 1 Henan Key Laboratory of Infrared Materials & Spectrum Measures and Applications, School of Physics, Henan Normal University, Xinxiang 453007, China; yukun@htu.edu.cn (K.Y.); shenpeng0720@126.com (P.S.); 15560168157@163.com (W.Z.); junzhang@htu.edu.cn (J.Z.); 2 School of Electrical Information Engineering, Henan University of Engineering, Zhengzhou 451191, China; goodxinan@126.com

**Keywords:** graphene, dual-band, metamaterial perfect absorber, surface plasmon

## Abstract

Due to its unique electronic and optical properties, graphene has been used to design tunable optical absorbers. In this paper, we propose a plasmonic absorber consisting of non-concentric graphene nanodisk arrays, which is designed to operate in the mid-infrared spectral range and is capable of achieving nearly perfect absorption. Two perfect absorption peaks are produced due to the impedance of the structure, which matches that of the free space. The influences of the thicknesses of the dielectric layer, the size of graphene nanodisk, and the incident conditions on the absorption are studied. Moreover, the absorption intensity can be independently tuned by varying the Fermi levels of two graphene nanodisks. Furthermore, the polarization-independent absorbance of the absorber exceeds 95% under oblique incidence, and remains very high over a wide angle. This proposed absorber has potential applications in optical detectors, tunable sensors, and band-pass filters.

## 1. Introduction

Perfect absorbers have a lot of important applications in the domain of photodetectors, filters, etc. [1,2]. Plasmonic metamaterials, which can support localized surface plasmons, are promising candidates for building perfect absorbers [3]. In recent years, metamatertial absorbers have mainly been focused on fixed-shaped noble metals [4]. However, noble metal has higher ohmic losses, and the absorption bands cannot be tuned independently from each other for multiple absorption peaks. Graphene, as a new ultra-thin optical material [5,6], is a two-dimensional carbon material with a single layer of carbon atoms. It has attracted considerable attention due to its unique optical and electronic properties [7,8,9], which support surface plasmons in infrared and terahertz bands. Compared to metallic nanoparticles, based on surface plasmons, graphene plasmons have much lower losses and stronger field confinements. More importantly, graphene plasmons have widely tunable electro-optical characters, and can be adjusted in real time with the external gate voltage or the chemical doping [10,11,12,13]. Therefore, graphene provides a new opportunity for tunable perfect absorbers.

A variety of graphene absorbers, such as ribbons, disks, and rings, have been investigated. For example, Meng et al. reported a periodically cross-shaped graphene ribbon array, where two absorber peak wavelengths can be simultaneously adjusted by tuning the Fermi energy of the graphene [14]. Li et al. demonstrated the periodic double-layer graphene ribbon arrays, where the single absorption peak wavelength can be tuned by a small change in the chemical potential of graphene [15]. Although these graphene absorbers have a tunable single or dual-band, most of them cannot be tuned independently. Sun et al. proposed a double-layer array of graphene nanodisks and nanoholes, and the dual-band absorption peak wavelengths can be tuned without varying the structure [16]. Wu et al. reported a triple-band infrared absorber that sustains three absorption peaks with independent tunability [17]. However, to date, few studies have focused on dual-band perfect absorbers in the mid-infrared range using graphene.

Herein, a simple graphene-based plasmonic absorber has been designed. The absorber consists of a gold mirror, dielectric layers, and dual-layer non-concentric graphene nanodisks. Through accurate simulations, we demonstrate two perfect absorption peaks due to the impedance of the structure matching that of the free space. The two absorption peaks originate from dipole resonances by the upper and lower nanodisks through the analysis of the electric field diagram, and can be independently controlled by varying the Fermi level and geometrical factor of the corresponding graphene layer. We further show that the absorber has the characteristic of polarization-insensitive absorption and maintains a high absorption over a wide range of incidence angles. This proposed absorber has potential applications in optical detectors, tunable sensors, and band-pass filters.

## 2. Materials and Methods

**Figure 1:**
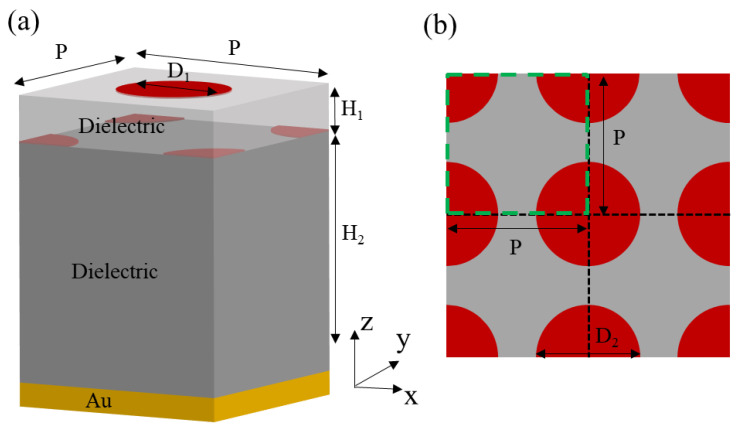
(**a**) Sketch of the proposed graphene-based absorber. (**b**) Top view of lower graphene nanodisk arrays.

Figure 1 shows the schematic view of the graphene-based infrared absorber. Double-layer graphene non-concentric nanodisk arrays are on the top of an optically thick gold layer. Arrays of graphene nanodisks with diameters of D_1_ = 150 nm and D_2_ = 180 nm are placed on the top surface of two dielectric layers with thicknesses of H_1_ = 60 nm and H_2_ = 1.5 μm, respectively. The periodicity P of the structure is 400 nm.

The two dielectric layers are regarded as lossless, and the permittivity is 1.96 [18]. The permittivity of gold follows the Drude model, with a plasma frequency of $\omega_{p}=1.37\times 10^{16}{\;s}^{-1}$ and damping constant of $\omega_{\tau }=1.23\times 10^{14}\;s^{-1}$ [19]. The conductivity of graphene includes inter-band transition and intra-band transition, which can be approximately expressed with the Kubo formula [20,21,22]

(1) $$\begin{array}{l} \sigma_{\omega }=\frac{2e^{2}k_{B}T}{\pi \hbar^{2}}\frac{i}{\omega +i\tau^{-1}}\ln\left[2\cosh\left(\frac{E_{F}}{2k_{B}T}\right)\right]+\\ \frac{e^{2}}{4\hbar }\left[\frac{1}{2}+\frac{1}{\pi }\arctan\left(\frac{\hbar \omega -2E_{F}}{2k_{B}T}\right)\right]-\frac{e^{2}}{4\hbar }\left[\frac{i}{2\pi }\ln\frac{{\left(\hbar \omega +2E_{F}\right)}^{2}}{{\left(\hbar \omega -2E_{F}\right)}^{2}+4{\left(k_{B}T\right)}^{2}}\right] \end{array}$$

where e corresponds to the elementary charge, $k_{B}$ to the Boltzmann constant, ℏ to the reduced Planck constant, T to the temperature, $E_{F}$ is the Fermi level, ω is the photon frequency in vacuum, and $\tau =\mu E_{F}/\left(ev_{F}^{2}\right)$ is the carrier relaxation time with the Fermi velocity $v_{F}=1\times 10^{6}{\;\mathrm{ms}}^{-1}$ and the DC mobility $\mu =10000{\;\mathrm{cm}}^{2}V^{-1}s^{-1}$ [23]. According to the Pauli incompatibility principle, we only considered the contribution of the electronic intra-band transition of graphene when the Fermi level $E_{F}>>k_{B}T$ and $E_{F}>>\hbar \omega$, so the Kubo formula can be approximately simplified the following Drude formula [23,24]

(2) $$\sigma_{\omega }=\frac{e^{2}E_{F}}{\pi \hbar^{2}}\frac{i}{\omega +i\tau^{-1}}$$

The numerical simulation is calculated by mature commercial COMSOL software (5.4, Stockholm, Sweden) based on the finite element method [25]. The absorptance can be written as *A* = 1 – *R* − *T*, where *R* and *T* are the reflectivity and transmittance, respectively. The gold substrate is thick enough so that the energy of the incident light cannot be transmitted. Thus, the absorption can be simplified as *A* = 1 − *R*. The perfect absorption *A* = 1 can be achieved when the impedance of the structure is matched with the free space impedance [26].

## 3. Results

**Figure 2:**
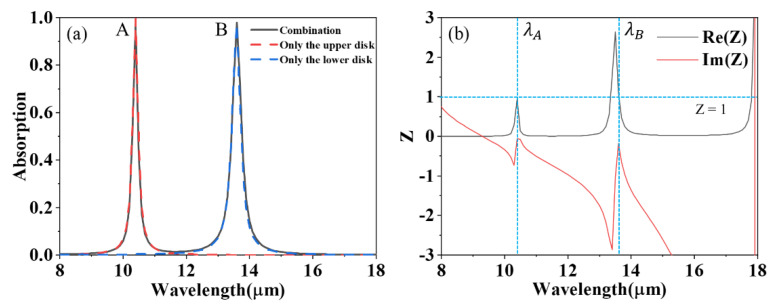
(**a**) The absorption spectrogram of non−concentric graphene−based absorber, where the red dashed line, blue dashed lines, and gray solid line represent the absorption of the upper nano−disk, lower nano−disk, and whole structure, respectively. (**b**) Variation diagram of normalized effective surface impedance with wavelength, where the gray curve and red curve are the real part and imaginary part of impedance, respectively. The horizontal blue dotted line indicates that the impedance value is 1, and the vertical blue dotted line is the corresponding position of absorption peaks A and B, respectively.

The absorption spectra of the graphene structure with the non-concentric bilayer are shown in Figure 2a. The grey-solid, red-dashed, and blue-dashed curves correspond to the absorption of the proposed absorber structure with the combination of two nanolayers, only a lower nanolayer, and only an upper nanolayer, respectively. The Fermi levels of the upper and lower nanodisks are set as 0.53 eV and 0.5 eV, respectively. It can be seen that the absorption spectra of the designed structure have two perfect absorption peaks at λ_A_ = 10.4 μm and λ_B_ = 13.6 μm. It can be seen the whole absorption spectra arises from the spectral superposition of the upper and lower graphene nanodisks, evidenced by the dashed line overlapping completely with the solid line. The physics of the structural perfect absorption can be understood by analyzing the normalized surface impedance [26]:

(3) $$\;Z=\sqrt{\frac{{\left(1+S_{11}\right)}^{2}-S_{21}^{2}}{{\left(1-S_{11}\right)}^{2}-S_{21}^{2}}},\;$$

where $S_{11}$ and $S_{21}\;$are used to express the reflection coefficient and transmission coefficient, respectively. Figure 2b shows the normalized impedance diagram. For the two absorption peaks, the real part of the effective impedance is close to 1, while the imaginary part is close to 0, which means the effective impedance of the structure can perfectly match the free space impedance and produce perfect absorption at the two resonance wavelengths.

**Figure 3:**
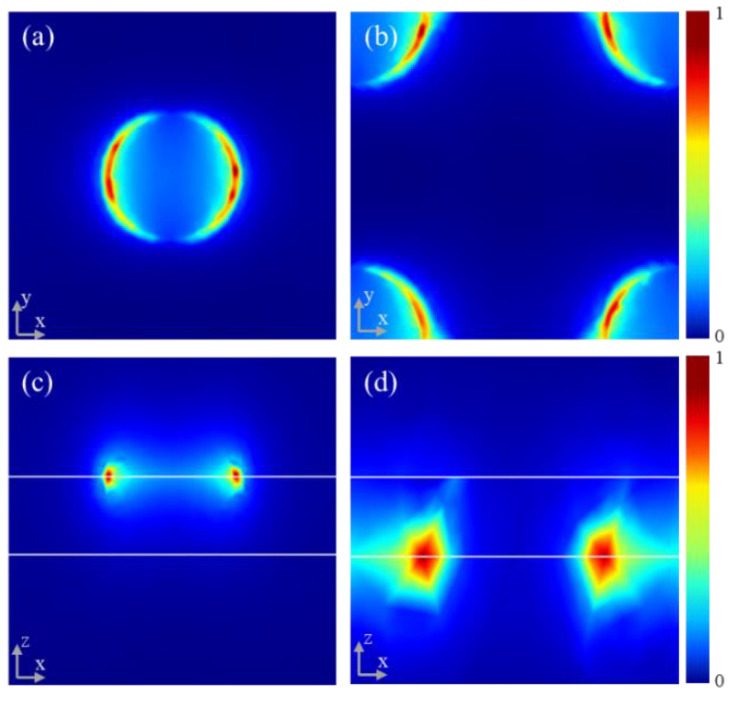
The normalized electric field intensity distribution (x-y incident plane) of (**a**) absorption peak A and (**b**) absorption peak B. The normalized electric field intensity distribution (x-z incident plane) of (**c**) absorption peak A and (**d**) absorption peak B.

Furthermore, we drew the normalized electric-field profiles of modes A (Figure 3a,c) and B (Figure 3b,d). Obviously, the electric field is highly symmetrically concentrated at the edge of the nanodisks, which indicates the two absorption peaks are from the contribution of the dipole resonance of the corresponding graphene nanodisk [27]. Furthermore, there is no near-field coupling between the double-layer graphene, which is another important element, giving rise to nearly perfect absorption.

**Figure 4:**
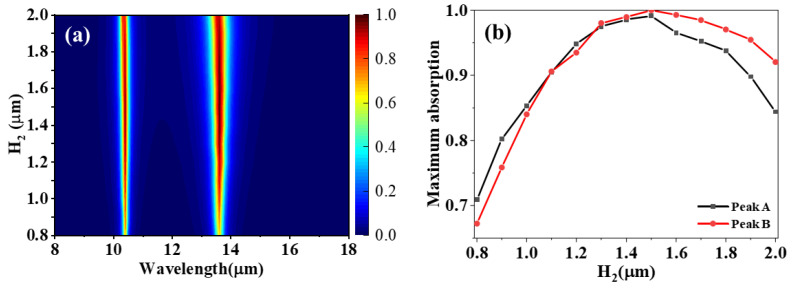
(**a**) The absorption spectra of the designed absorber and (**b**) the maximum absorption of two absorption peaks as a function of H_2_.

The suitable thickness of the dielectric layer is the key factor for matching the impedance of the absorber. In order to realize efficient absorption of the incident waves, the impedance of the absorber should be perfectly matched with the free space impedance [26]. Absorption spectra as a function of the thickness H_2_ of the lower dielectric layer is studied. As can be seen from Figure 4a, the positions of the two absorption peaks are basically unchanged with the increase of thickness H_2_ of the lower dielectric layer. The reason for this is Fabry–Perot effects in the second insulator layer with thickness H_2_. Figure 4b shows the relationship between the maximum absorption of peaks A and B and the thickness H_2_ of the lower dielectric layer. It is found that the maximum absorption of peaks A and B increase first and then decrease with the increase in the thickness H_2_, and both reach the maximum absorption at H_2_ = 1.5 μm. Thus, in this work, H_2_ = 1.5 μm is selected, and a lot of incident light are absorbed because the impedance of the absorber and the free space is matched perfectly at this thickness.

**Figure 5:**
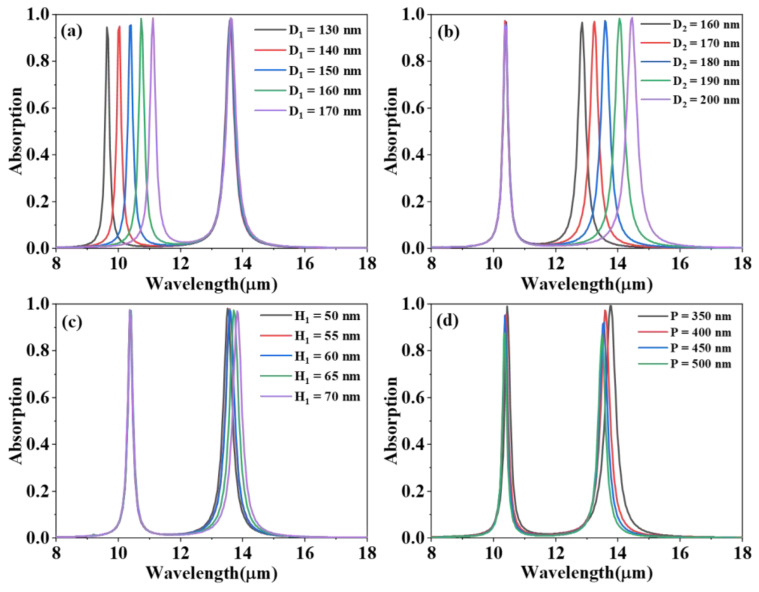
Absorption spectra of the proposed absorber for different (**a**) diameters D_1_ of the upper graphene nanodisk, (**b**) diameters D_2_ of the lower graphene nanodisk, (**c**) thickness H_1_ of the upper dielectric, and (**d**) the period P of the structure.

In addition, the influence of the geometric parameters of the proposed absorber is analyzed carefully. Figure 5 shows the absorption spectrum of the proposed absorber for different structural parameters. From Figure 5a it can be seen that the absorption peak A has a red-shift from 9.64 μm to 11.10 μm when the diameter D_1_ of the upper graphene nanodisk changes from 130 nm to 170 nm. At the same time, the absorption peak B does not move at all. However, in Figure 5b, absorption peak B has a red-shift from 12.82 μm to 14.45 μm when the diameter D_2_ of the lower graphene nanodisk changes from 160 nm to 200 nm, and absorption peak A still remains unchanged. The following principles can be used to explain the phenomenon. Plasmon resonance occurs only when the energy of a single graphene nanodisk satisfies the following relationship [28,29]

(4) $$\hbar \omega_{p}\approx \sqrt{\frac{2\alpha \hbar cL_{1}E_{F}}{\pi \left(\varepsilon_{1}+\varepsilon_{2}\right)D}}$$

where $\alpha =e^{2}/4\pi \varepsilon_{0}\hbar c\approx 1/13$ accounts for the fine structure constant, ℏ is the reduced Planck constant, and c indicates the light speed in vacuum. *L*_1_ is a constant associated with the symmetry of plasmons; $\varepsilon_{1}$ and $\varepsilon_{2}$ are the permittivity of the dielectrics above and below the graphene, respectively; and *D* is the diameter of the graphene nanodisk. Therefore, we can obtain the corresponding resonance wavelength from Equation (4), as follows:

(5) $$\lambda_{p}\approx \sqrt{\frac{2\pi^{3}c\hbar \left(\varepsilon_{1}+\varepsilon_{2}\right)D}{\alpha L_{1}E_{F}}}$$

Evidently, the resonant wavelength has a positive correlation with the diameter of the graphene disk. Therefore, absorption peaks A and B will red-shift with the increase of the diameter of the corresponding graphene nanolayers, which is in good agreement with the simulation in Figure 5a,b.

As seen from Figure 5c, the two absorption peaks have almost no major changes with increasing H_1_ from 50 nm to 70 nm. The main reason for this is that the thickness of dielectric H_1_ is far less than the operation wavelength of the structure, and the Fabry–Pérot effect related to the top dielectric layer can be effectively prevented.

Figure 5d shows the effect of different periods P on the absorption spectrum. The absorption peak A has only a tiny shift from 10.44 μm to 10.36 μm with the change of period P from 350 nm to 500 nm. The effective dielectric constant of the insulating dielectric layer is slightly reduced because of the increase of P at constant D_2_ [16]. According to Equation (5), the absorption wavelength will blue-shift when the dielectric constant decreases. Compared to absorption peak A, absorption peak B has a relatively obvious blue-shift from 13.76 μm to 13.50 μm when P increases from 350 nm to 500 nm. This is because *L*_1_ is increased as the ratio of the inner diameter to outer diameter of the ring decreases [28]. According to Equation (5), the resonance wavelength naturally decreases as *L*_1_ increases, thus absorption peak B blue-shifts.

**Figure 6:**
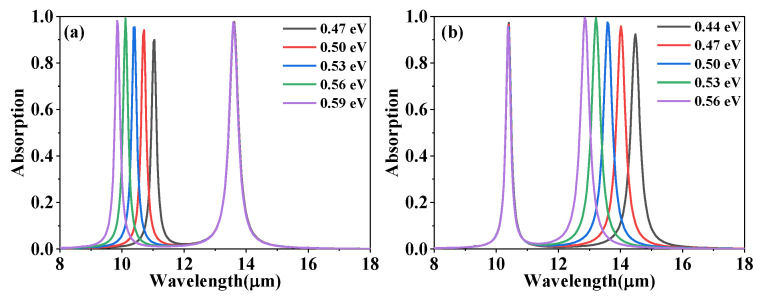
The absorption spectra of the designed absorber with Fermi levels for (**a**) E_F1_ of the upper graphene nanodisk and (**b**) E_F2_ of the lower graphene nanodisk.

It is known that the absorption peaks of the designed absorber can be independently tuned by varying the Fermi level of the corresponding graphene layer. Figure 6a reveals the absorption spectra with different Fermi levels E_F1_. Peak A experiences a blue-shift from 11.04 μm to 9.83 μm as the Fermi level E_F1_ varies from 0.47 eV to 0.59 eV, while peak B remains unchanged. Figure 6b shows the absorption spectra changes with different Fermi levels E_F2_. Contrary to Figure 6a, peak B carries out a blue-shift from 14.48 μm to 12.85 μm with the increase in the Fermi level E_F2_ from 0.44 eV to 0.56 eV, whereas peak A remains constant. The absorption peaks shift to blue with the increase in the Fermi levels of the corresponding graphene nanodisk, which can be explained as follows: The shift of resonance frequency is determined by the imaginary part of graphene conductivity. When the Fermi level increases, the imaginary part of graphene conductivity increases [26], resulting in the red shift of the resonance frequency (namely resonance wavelengths shift to blue). Moreover, the near-field coupling effect does not take place between the two absorption peaks, and the absorption spectrum can be independently adjusted by only changing the Fermi level of the corresponding graphene nanolayers. In addition, as can be seen from Figure 6a,b, the two largest absorption peaks occur at E_F1_ = 0.56 eV and E_F2_ = 0.53 eV, respectively, where the impedance of the absorber is perfectly matched with the impedance of the free space [26]. Compared with the absorber with the metal structure, the absorber based on graphene is capable of independently and freely adjusting the absorption characteristics without the need for changing the structural parameters.

**Figure 7:**
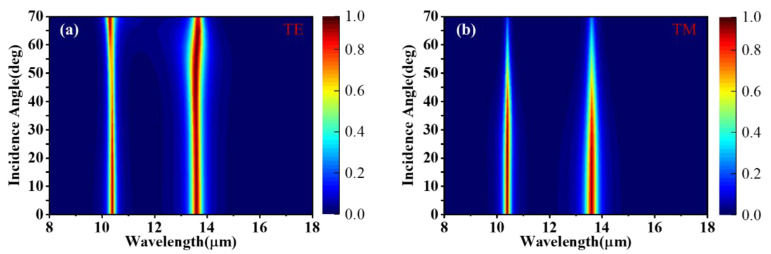
Absorption spectra for (**a**) transverse electric wave (TE) polarization and (**b**) transverse magnetic wave (TM) polarization as a function of the incidence angle.

The case of different polarized light incident obliquely is discussed in Figure 7a,b, where we calculate the angle-resolved absorption spectra of the structure under TE and TM polarized light, respectively. It can be intuitively seen that even if the incident angle is large, the spectral position of the absorption peaks remains almost unchanged, and two perfect absorption peaks can still be realized. Because the structure is highly symmetrical, the absorption peak is not sensitive to the incident angle. The designed absorber has the characteristics of independent adjustment, polarization independence, and wide-angle.

## 4. Conclusions

A simple structure for an independently tunable dual-band perfect infrared absorber based on non-concentric graphene nanodisk has been demonstrated. The two absorption peaks originate from the dipole resonance mode formed by the corresponding graphene nanodisk. Moreover, the near-unity absorption is because the impedance of the structure matches the impedance of the free space at the wavelength corresponding to the absorption peak. We analyzed the influence of the structural parameters on the absorption spectra. It is shown that the absorption strength can be independently tuned by varying the Fermi levels of graphene in the corresponding nanodisks. Furthermore, two perfect absorption peaks can be obtained over a wide incident angle span, and are insensitive to the incident angle for TE and TM polarizations. This proposed infrared absorber has potential applications in optical detectors, tunable sensors, and band-pass filters.

## Data Availability

Data underlying the results presented in this paper are not publicly available at this time, but may be obtained from the authors upon reasonable request.
